# Hydroxysafflor Yellow A Exerts Neuroprotective Effects via HIF-1α/BNIP3 Pathway to Activate Neuronal Autophagy after OGD/R

**DOI:** 10.3390/cells11233726

**Published:** 2022-11-22

**Authors:** Ruheng Wei, Lijuan Song, Zhuyue Miao, Kexin Liu, Guangyuan Han, Haifei Zhang, Dong Ma, Jianjun Huang, Hao Tian, Baoguo Xiao, Cungen Ma

**Affiliations:** 1 The Key Research Laboratory of Benefiting Qi for Acting Blood Circulation Method to Treat Multiple Sclerosis of State Administration of Traditional Chinese Medicine, Jinzhong 030619, China; 2 Research Center of Neurobiology, Shanxi University of Chinese Medicine, Jinzhong 030619, China; 3 Department of Physiology, Shanxi Medical University, Taiyuan 030001, China; 4 Department of Neurology, First Affiliated Hospital, Shanxi Datong University, Datong 037009, China; 5 Shanxi Health Commission Key Laboratory of Nervous System Disease Prevention and Treatment, Datong 037003, China; 6 Institute of Neurology, Huashan Hospital, Fudan University, Shanghai 200433, China; 7 State Key Laboratory of Medical Neurobiology, Fudan University, Shanghai 200433, China; 8 Institute of Brain Science, Shanxi Datong University, Datong 037008, China

**Keywords:** HSYA, OGD/R, HIF-1α/BNIP3 pathway, autophagy, apoptosis

## Abstract

In the process of ischemic stroke (IS), cellular macroautophagy/autophagy and apoptosis play a vital role in neuroprotection against it. Therefore, regulating their balance is a potential therapeutic strategy. It has been proved that hydroxysafflor yellow A (HSYA) has anti-inflammatory and antioxidant effects, which can both protect neurons. By exploring bioinformatics combined with network pharmacology, we found that HIF1A and CASP3, key factors regulating autophagy and apoptosis, may be important targets of HSYA for neuroprotection in an oxygen glucose deprivation and reperfusion (OGD/R) model. In this study, we explored a possible new mechanism of HSYA neuroprotection in the OGD/R model. The results showed that OGD/R increased the expression of HIF1A and CASP3 in SH-SY5Y cells and induced autophagy and apoptosis, while HSYA intervention further promoted the expression of HIF1A and inhibited the level of CASP3, accompanied by an increase in autophagy and a decrease in apoptosis in SH-SY5Y cells. The inhibition of HIF1A diminished the activation of autophagy induced with HSYA, while the inhibition of autophagy increased cell apoptosis and blocked the neuroprotective effect of HSYA, suggesting that the neuroprotective effect of HSYA should be mediated by activating the HIF1A/BNIP3 signaling pathway to induce autophagy. These results demonstrate that HSYA may be a promising agent for treating IS.

## 1. Introduction

Ischemic stroke (IS) is a disease caused by reduced brain blood flow or complete occlusion of cerebral blood flow, resulting in corresponding neurological deficits [1]. Ischemic stroke not only has a high rate of disability and mortality but also causes sequelae and complications that seriously affect the life quality of patients and are a social and economic burden worldwide [2]. Cerebral ischemia/reperfusion (I/R) injury is an important pathophysiological alteration in the disease, which can produce adverse effects such as vascular endothelial damage [3] and impaired neuronal function [4], and seriously affect the outcome of stroke. Although the US Food and Drug Administration (FDA) approved the application of thrombolytic agents for IS, the narrow therapeutic time window and risk of cerebral hemorrhage limit the widespread use of recombinant plasminogen activators [5]. Therefore, there is an urgent need to focus on potential therapeutic strategies for IS.

Hydroxysafflor yellow A (HSYA) is a glycoside belonging to the monochalcone class of the compounds and is the most potent water-soluble monomeric component in the traditional Chinese medicine saffron [6]. The potential mechanisms of HSYA as an effective agent in the treatment of IS may be related to a variety of biological processes, including the reduction in infarct volume and brain oedema, the inhibition of inflammation and oxidation, the inhibition of thrombosis and platelet aggregation, and the protection of blood vessels and neurons [7,8,9]. However, the cellular and molecular mechanisms of HSYA action in IS are relatively lacking and need to be further elucidated.

By using bioinformatics approaches, we initially screened genes that play a critical regulatory role between the pathogenesis of IS and the functions of HSYA, providing potential cellular and molecular targets for HSYA action [10,11]. To verify the information from the analysis of the bioinformatics approaches, we constructed an in vitro neuronal cell (SH-SY5Y) OGD/R injury model and intervened with HSYA to test the related hypotheses. Our results will help to reveal effective targets related to IS and provide an experimental basis for the neuroprotective mechanism of HSYA action in IS.

## 2. Materials and Methods

### 2.1. Data Collection and Pre-Processing

The Gene Expression Omnibus (GEO) database (http://www.ncbi.nlm.nih.gov/geo, accessed on 28 March 2022) [12] was used to obtain gene expression profiles for IS by searching for the keyword “ischemic stroke”. The GSE100235 microarray dataset with good variability was selected for the study’s next step. The data included the sham group and the middle cerebral artery occlusion (MCAO) model group. We used the R package “affy” for normalization and background correction of the data. The “limma” package (version 3.40.6) was used for differential analysis to obtain differential genes between the model and control groups. Specifically, we obtained expression profile datasets by first targeting probes to genes and removing null probes. If multiple probes were localized to the same gene, its mean value was selected as the expression level of that gene. Finally, the differential significance of each gene was obtained. Gene expression values of the |log2 FC| > 1 and adjusted *p* < 0.05 were used for filtering differentially expressed genes (DEGs).

### 2.2. Identification of IS-Related Differentially Expressed Genes

Genes were retrieved from the National Center for Biotechnology Information (NCBI) platform (https://www.ncbi.nlm.nih.gov/, accessed on 28 March 2022) and GengCards database (www.genecards.org/, accessed on 28 March 2022) [13] with the keyword “cerebral ischemic stroke”, and the species “Homo sapiens” intersected the stroke-related genes collected from both platforms with DEGs. We finally obtained IS-related differential genes (IS-DEGs) co-expressed with human attributes and further analyzed them.

### 2.3. Identification of Protein–Protein Interaction (PPI) Networks of IS-DEGs

The PPI interaction network of IS-DEGs was constructed by using the online database STRING (https://www.string-db.org/, accessed on 29 March 2022) with an interaction score > 0.4. The results of this analysis were downloaded and applied to Cytoscape software (V3.8.0) for visual analysis, and the hub genes were screened by the Matthews correlation coefficient (MCC) algorithm of the cytoHubba-v0.1 plugin.

### 2.4. Functional Enrichment Analysis of Differential Genes

Gene Ontology (GO) and Kyoto Encyclopedia of Genes and Genomes (KEGG) analyses were performed using the online tool, the Database for Annotation, Visualization, and Integrated Discovery (DAVID) (https://david.ncifcrf.gov/, accessed on 31 March 2022), with the results annotated and visualized.

### 2.5. Therapeutic Target Screening of HSYA for IS

The chemical structural formula of HSYA and the related target information were retrieved from the Traditional Chinese Medicine Systems Pharmacology database (TCMSP) (http://lsp.nwsuaf.edu.cn/tcmsp.php, accessed on 6 April 2022) [14]. The gene names and IDs of HSYA targets were further extracted using UniProt (http://www.uniprot.org, accessed on 6 April 2022) [15], an authoritative database of protein sequences intersected with IS-DEGs and validated by in vitro experiments.

### 2.6. Cell Culture

SH-SY5Y cells, human neuroblastoma cells, were obtained from the cell bank of Chinese Academy of Sciences (Shanghai, China). Cells were routinely cultured in Dulbecco’s Modified Eagle’s Medium (DMEM, Gibco, Clayton, Australia) containing 10% fetal bovine serum (FBS, Gibco, Clayton, Australia), 10 U/mL penicillin, and 10 U/mL streptomycin, and were maintained at 37 °C in a humidified atmosphere containing 5% CO_2_, with medium changes every 2 days. An in vitro model of I/R was established in SH-SY5Y cells.

### 2.7. OGD/R and Agent Treatments

We used the OGD/R model to simulate in vitro ischemia/reperfusion (I/R)-like conditions. First, after discarding the complete medium and washing the cells with PBS, DMEM without glucose was added to the cells and placed in an anaerobic incubator (0.1% O_2_, 5% CO_2_, 94% N_2_, and 37 °C) for 6 h. After the OGD period, glucose was supplemented to normal levels. The cells were then placed in an incubator with normal growth conditions (37 °C containing 5% CO_2_ and 95% O_2_) and continued to incubate for 12 h. HSYA (25 μmol/L) and autophagy inhibitor 3-Methyladenine (3-MA) (5 mmol/L) were simultaneously administered. Hypoxia-inducible factor 1alpha (HIF-1α) inhibitor 3-(5′-Hydroxymethyl-2′-furyl)-1-benzylindazole (YC-1) was pretreated with 10 μM for 2 h prior to OGD.

### 2.8. Transmission Electron Microscope Analysis (TEM)

The treated SH-SY5Y cells were digested with trypsin and centrifuged at 1500 rpm for 5 min. Subsequently, cell precipitates were collected, double fixed, dehydrated, infiltrated, embedded, ultrathin sectioned, stained, and observed under the TEM for cell morphology.

### 2.9. Western Blot Analysis

SH-SY5Y cells were discarded from the complete medium, washed 3 times with PBS, and lysed for 30 min with cell lysis buffer containing 1 mM PMSF (Beyotime) and phosphatase inhibitor for Western blot (WB) and immunoprecipitation (IP). Quantitative protein samples were then separated via sodium dodecyl sulfate-polyacrylamide gel electrophoresis and blotted onto polyvinylidene difluoride membranes. The membranes were blocked for 1 h at room temperature (RT) using 5 % skim milk and then incubated separately with primary antibodies at 4 °C overnight. The next day, the membranes were washed 3 times (10 min) with TBST and incubated with a secondary antibody for 45 min at 37 °C. The immunoreactive bands were detected using BeyoECL Plus (Beyotime).

### 2.10. mCherry-GFP-LC3B Adenovirus Transfection

SH-SY5Y cells were inoculated into 24-well plates and transfected with Ad-mCherry-GFP-LC3B for 24 h. The multiplicity of infection (MOI) was determined. Subsequently, the pre-transfected cells were subjected to OGD/R treatment, and HSYA and 3-MA interventions were added. Finally, the mCherry-GFP-LC3B fusion protein was visualized by fluorescence microscopy.

### 2.11. TUNEL Staining

According to the manufacturer’s instructions, apoptosis was determined using a terminal deoxynucleotidyl transferase-mediated dUTP nick end labeling (TUNEL) kit (Biyuntian Biotechnology Co., Ltd., Beijing, China). Briefly, after discarding the culture medium and rinsing three times with PBS, the cells were fixed in 4% paraformaldehyde for 30 min, then permeabilized with PBS containing 0.3% Triton X-100 for 15 min and incubated with TUNEL assay solution for 1 h at 37 °C. The nuclei of TUNEL-positive cells were observed by fluorescence microscope and were counted using Image-Pro Plus 5.0 (Media Cybernetics Inc., Rockville, MD, USA). The experiments were repeated 3 times independently.

### 2.12. Flow Cytometric Evaluation of Apoptosis (Annexin-V/PI)

Annexin V (Annexin V-FITC) and propidium iodide (PI) Apoptosis Detection Kit (Beyotime) was used to detect apoptosis in SH-SY5Y cells. Briefly, SH-SY5Y cells were collected after various treatments and resuspended in 195 μL Annexin V-FITC binding buffer. Then, 5 μL AnnexinV-FITC and 10 μL PI solution were added to SH-SY5Y cell buffer and incubated at RT in the dark for 15 min. Apoptosis was detected on a flow cytometer. Data were collected from at least 10 thousand cells in three independent experiments.

### 2.13. Statistical Analysis

Analysis was performed using GraphPad Prism 8.0 software (USA). All values are expressed as mean ± standard deviation. One-way ANOVA and post hoc least significant difference multiple comparison tests were used to compare more than two groups. *p* < 0.05 was considered statistically significant.

## 3. Results

### 3.1. Identification of DEGs and IS-Related DEGs

**Figure 1:**
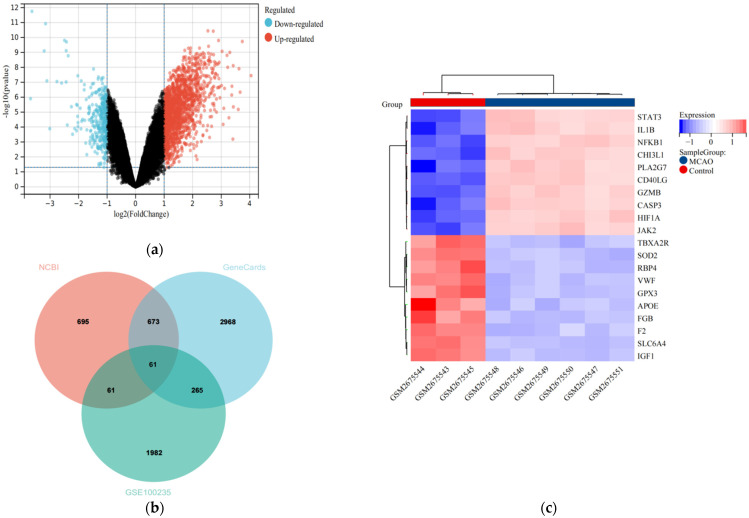
Identification of DEGs and Ischemic Stroke-Related DEGs. (**a**) Volcano plot of DEGs between the sham group and the MCAO samples. The red plots represent upregulated genes, the black plots represent nonsignificant genes, and the blue plots represent downregulated genes. (**b**) The genes from three sources were taken to intersect to obtain IS-DEGs. (**c**) Heatmap of first 20 DEGs between the sham group and the MCAO samples. Red rectangles represent high expression, and blue rectangles represent low expression.

Gene expression data GSE100235 was downloaded from GEO, 19,634 genes were identified, and the differences between model and control groups in the dataset were analyzed using the limma package in R. We identified 1982 DEGs, including 1613 upregulated and 369 downregulated (Figure 1a). In addition, a total of 695 human IS-related genes were obtained on the NCBI platform, and 3000 stroke genes with a score > 1 were selected in the GengCards database. Finally, the genes obtained from both platforms were intersected with the DEGs to obtain 61 IS-DEGs (Figure 1b,c).

### 3.2. PPI Network Analysis of IS-DEGs, and Identification of Hub Genes

**Figure 2:**
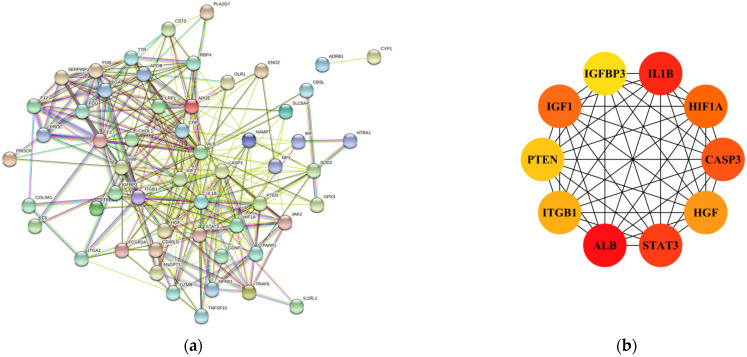
PPI network analysis of the IS-DEGs identified. (**a**) PPI network of all the IS-DEGs (interaction score >0.4). (**b**) The interaction network of top 10 hub genes.

In the following, 61 IS-DEGs were imported into the STING database for PPI network construction, and the analysis results were obtained according to the interaction score of >0.4 (Figure 2a). Next, we used Cytoscape software to visualize the above results and obtained the top ten ranked hub genes by the MCC algorithm of the cytoHubba-v0.1 plugin (Figure 2b).

### 3.3. GO and KEGG Enrichment Analysis

**Figure 3:**
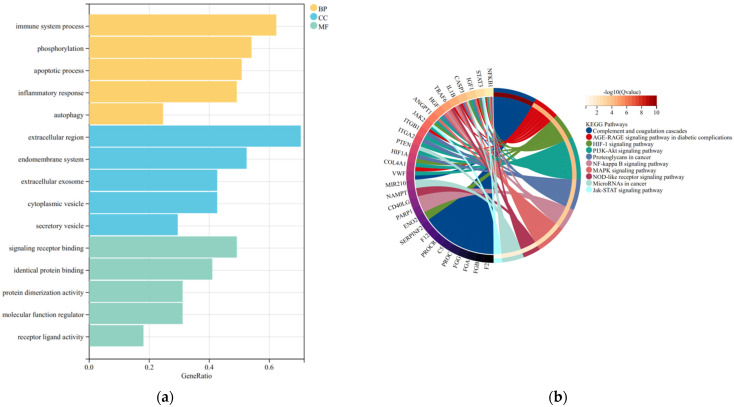
GO and KEGG pathway enrichment analyses of IS-DEGs. (**a**) The bubble plot showing the three aspects of enriched biological processes of biological process (BP), cell component (CC), and molecular function (MF), such as immune response and inflammatory response. (**b**) The chord plot showing the top 10 most enriched KEGG pathways of IS-DEGs. The screening criterion for significantly enriched biological processes and pathways was Q < 0.05. The Q value is the adjusted p value.

Using DAVID and visualization, we obtained the main biological processes involved in IS-DEGs. GO analysis included biological process (BP), cellular component (CC), and molecular function (MF). The results showed the correlation with immune response, inflammatory response, phosphorylation, and apoptosis (Figure 3a). The effects of KEGG pathway analysis are shown in Figure 3b. AGE-RAGE signaling pathway, HIF-1 signaling pathway, and PI3K-Akt signaling pathway are involved in regulating the process of IS.

### 3.4. Target Prediction and Experimental Validation of HSYA for the Treatment of IS

**Figure 4:**
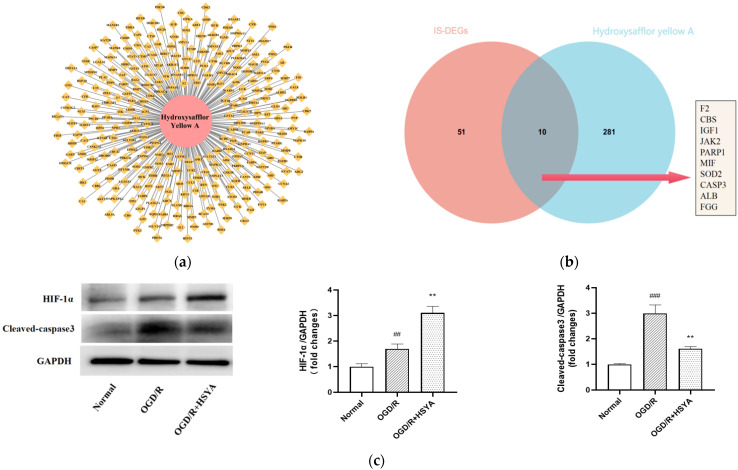

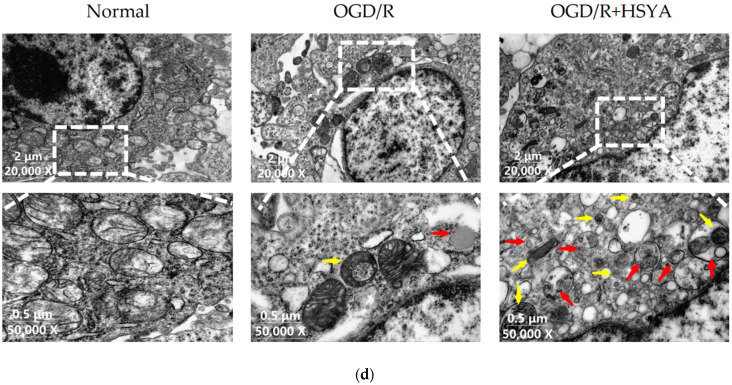
Screening the therapeutic target network of HSYA and selecting pivotal genes for validation. (**a**) Therapeutic targets of HSYA extracted using TCMSP. (**b**) Potential target genes for HSYA treatment of IS. (**c**) Western blotting was performed to detect the expression of HIF-1α and Cleaved-Caspase-3. GAPDH was used as an internal control. (**d**) Images of OGD/R-damaged SH-SY5Y cells obtained from TEM. Yellow arrows indicate autophagosomes and red arrows indicate autolysosomes. The images in the lower panel are the enlarged areas of the rectangular boxes in the upper panel. The image is one of the representative results for each group. Results are representative of three independent experiments, and the data were statistically analyzed using one-way ANOVA and expressed as mean ± standard deviation. (Note: compared with the normal group, ### *p* < 0.001, ## *p* < 0.01; compared with the OGD/R group, ** *p* < 0.01.)

The HSYA-related chemical structural formulae were obtained from the TCMSP database, and 291 target genes (Figure 4a) for HSYA were obtained after gene ID transformation through the UniProt database. Combined with IS-DEGs, ten potential target genes of HSYA were obtained, specifically F2, CBS, IGF1, JAK2, PARP1, MIF, SOD2, CASP3, ALB, and FGG (Figure 4b). By combining the prediction results with literature reports, we selected HIF1A and CASP3 as target genes for exploring the mechanism of HSYA action in the treatment of experimental IS. 

Next, we determined the optimal concentration of HSYA at 25 μmol/L for subsequent experiments (Supplementary Figure S1a,b). WB results revealed that the expression of both HIF1A and CASP3 was upregulated in OGD/R-injured SH-SY5Y cells, which was consistent with our predicted results. In addition, HSYA intervention further increased HIF1A expression and decreased CASP3 expression compared to the OGD/R group (Figure 4c). Typical apoptotic and necrotic features were observed in the cells under the TEM. Interestingly, cells in the OGD/R group started to display vesicles with autophagosomal characteristics, suggesting that OGD/R caused the activation of autophagy, which was further increased by HSYA treatment in OGD/R-injured SH-SY5Y cells (Figure 4d), Based on this phenomenon, we further investigated the potential relationship between autophagy and HSYA action.

### 3.5. HSYA Treatment Increases Autophagy in OGD/R-Injured SH-SY5Y Cells

**Figure 5:**
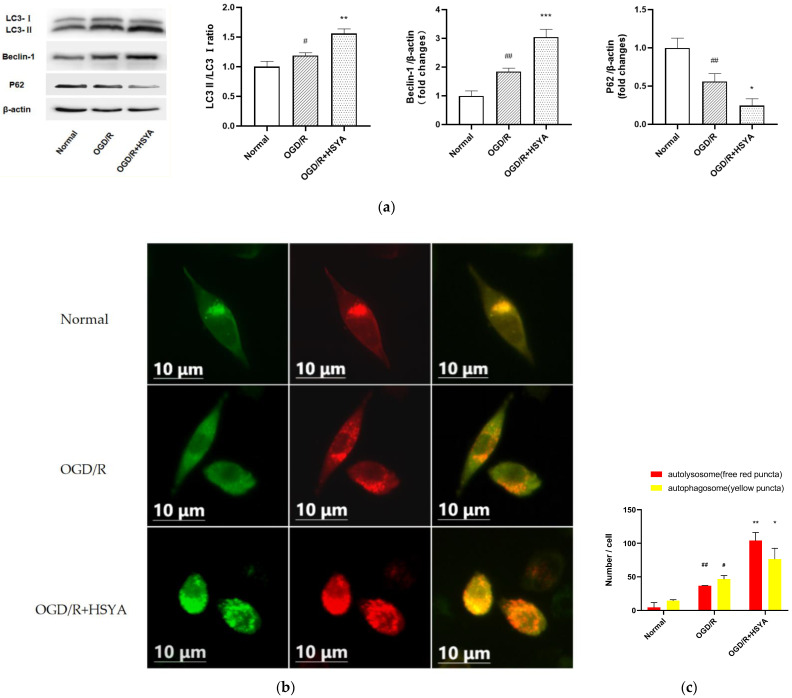
HSYA activates autophagy and maintains a stable autophagic flux in SH-SY5Y cells during the acute phase of OGD/R injury. (**a**) Western blotting for the levels of LC3, Beclin-1, and p62 in SH-SY5Y cells treated with indicated conditions. (**b**) Changes in autophagic flux of HSYA on OGD/R-treated SH-SY5Y cells. Fluorescence microscopy analysis of SH-SY5Y cells transfected with mCherry-GFP-LC3B; the image shown is one of the representative results for each group. (**c**) Statistical analysis of fluorescent dots in SH-SY5Y cells. Yellow dots indicate autophagosomes and red dots indicate autolysosomes in the merged images. Results are representative of three independent experiments, and the data were statistically analyzed using one-way ANOVA and expressed as mean ± standard deviation. (Note: compared with the normal group, ## *p* < 0.01, # *p* < 0.05; compared with the OGD/R group, *** *p* < 0.001,** *p* < 0.01, * *p* < 0.05.)

Autophagy, as an intracellular degradation/reuse pathway, plays a vital role in the course of IS. In our study, to assess the effect of HSYA on the autophagy of neurons, we observed autophagy-related indicators LC3, Beclin-1, and P62. WB results showed that LC3II/LC3I and Beclin-1 expression increased and P62 expression decreased in SH-SY5Y cells after OGD/R, while HSYA intervention further increased these trends (Figure 5a). To confirm that HSYA-activated autophagy regulates the balance of autophagic flux, we transfected cells with tandem mCherry-GFP-LC3B adenovirus and observed changes in the autophagosomes and autophagolysosomes by fluorescence microscopy. Given that mCherry fluorescence remains even under acidic conditions in the lysosome lumen where GFP loses its fluorescence, the yellow spots in the merged images (co-localization of GFP and mCherry fluorescence) represent autophagosomes. In contrast, the red spots only indicate autolysosomes. The change in the number of red and yellow spots represents an alteration in autophagic flux. The results showed a small number of red and yellow spots in the OGD/R model, representing a slight activation of autophagic flux. There was a significant increase in red and yellow spots after HSYA intervention, which further activated the autophagic flux (Figure 5b,c).

### 3.6. HSYA Attenuates Apoptosis by Regulating Autophagy in OGD/R-Injured Neurons

**Figure 6:**
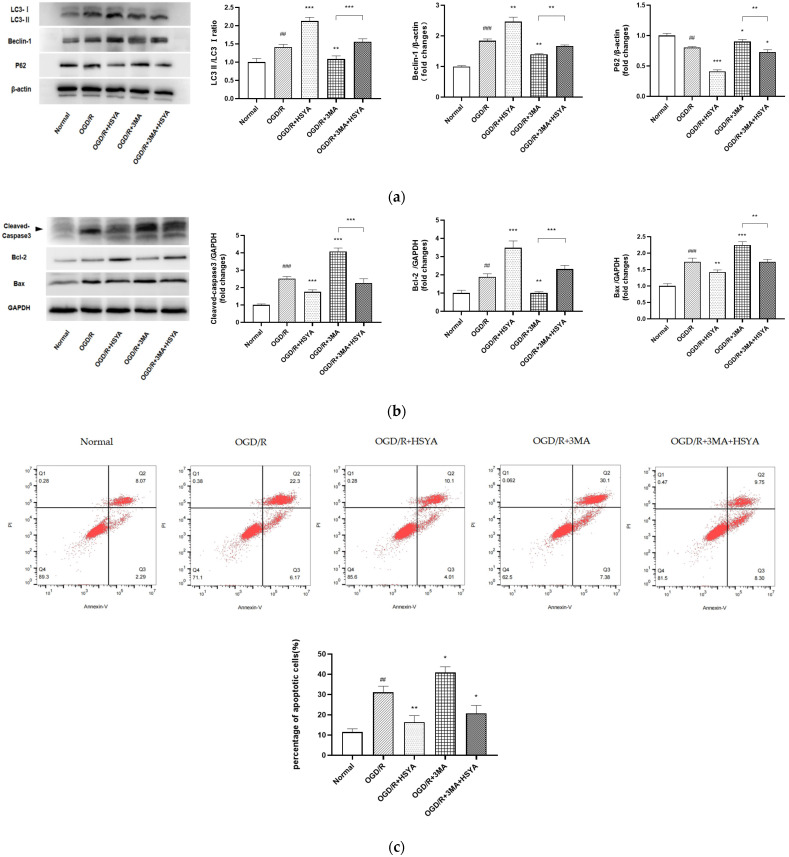
Enhanced autophagic activity mediates the protective role of HSYA in OGD/R cytotoxicity. (**a**) Western blotting was performed to detect the expression of LC3, Beclin-1, and P62. β-actin was used as an internal control. (**b**) Western blotting was performed to detect the expression of Bcl-2, BAX, and Cleaved-Caspase-3. GAPDH was used as an internal control. (**c**) Flow cytometry detection of Annexin V-FITC/PI double-stained cells in different treatment groups. Staining shows that HSYA reduces the percentage of OGD/R-induced apoptotic SH-SY5Y cells. The upper and lower right quadrants of the flow cytometry plot depict late apoptosis and early apoptosis, respectively. Usually, the total percentage of early and late apoptotic cells is used to indicate the total apoptotic cells. Results are representative of three independent experiments, and the data were statistically analyzed using one-way ANOVA and expressed as mean ± standard deviation. (Note: compared with the normal group, ### *p* < 0.001, ## *p* < 0.01; compared with the OGD/R group, *** *p* < 0.001, ** *p* < 0.01, * *p* < 0.05.)

Next, different methods were used to verify autophagy’s effect on cell apoptosis. First, the autophagic activation after HSYA intervention was accompanied by the survival of SH-SY5Y cells by 3-(4,5-dimethylthiazol-2-yl)-2,5-diphenyltetrazolium bromide (MTT) and lactate dehydrogenase (LDH) assays (Supplementary Figure S2a,b). In addition, to verify whether HSYA-mediated autophagy participates in cell apoptosis during OGD/R injury, we used the autophagy inhibitor 3MA to evaluate autophagy changes (LC3II/LC3I ratio, Beclin-1 and P62 expression) and apoptosis-related indicators (Cleaved-Caspase-3 (Cl-Casp3), BAX, and Bcl-2). The results showed that the inhibition of autophagy increased the expression of Cl-Casp3 and BAX and decreased the expression of Bcl-2 (Figure 6a,b). Further, the flow cytometry results also showed that the inhibition of autophagy blocked the neuroprotective effect of HSYA (Figure 6c), suggesting that the neuroprotective effect of HSYA is related to the activation of autophagy during OGD/R.

### 3.7. HIF-1α/BNIP3 Signaling Pathway Regulates HSYA-Induced Autophagy to Protect Neurons

**Figure 7:**
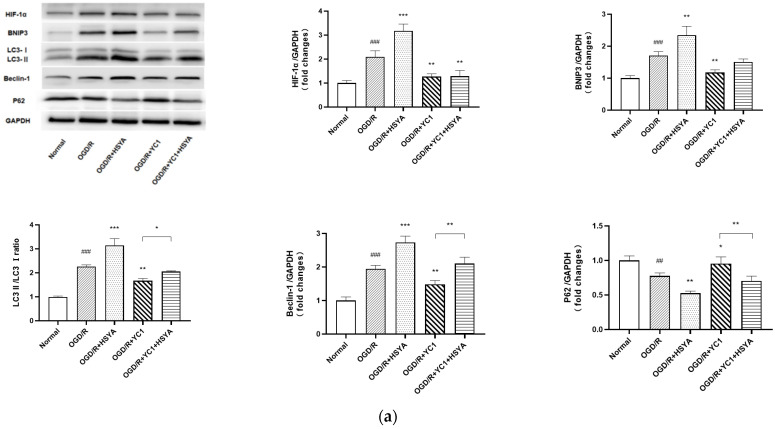

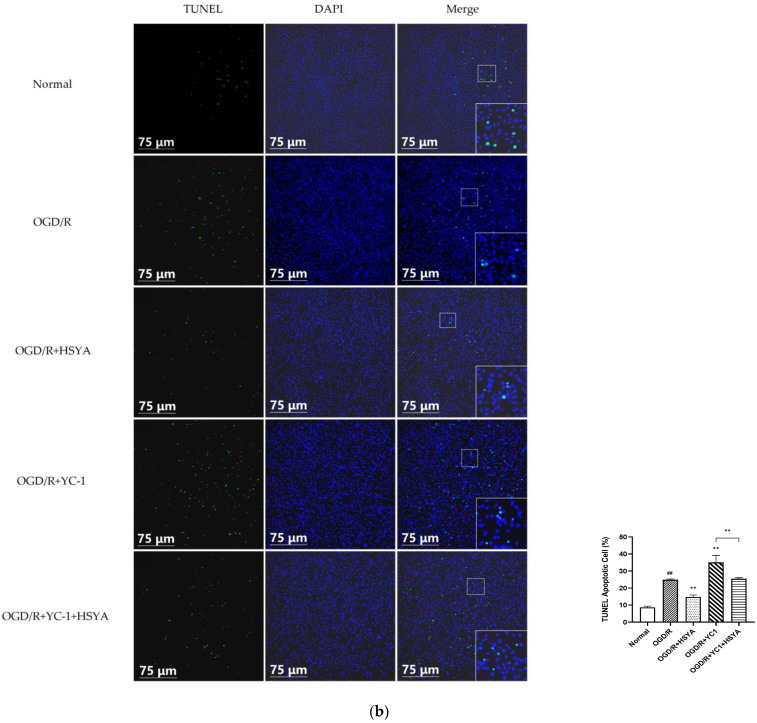
HIF-1α/BNIP3 pathway regulates HSYA-induced autophagy in neuroprotection after OGD/R. (**a**) Western blotting was performed to detect the expression of LC3B, Beclin-1, P62, Bcl-2, BAX, and Cleaved-Caspase-3 after YC-1 pretreatment. GAPDH was used as an internal control. (**b**) Representative images of TUNEL assays are shown. TUNEL-positive cells are green; nuclei are blue. Results are representative of three independent experiments, and the data were statistically analyzed using one-way ANOVA and expressed as mean ± standard deviation. (Note: compared with the normal group, ### *p* < 0.001, ## *p* < 0.01; compared with the OGD/R group, *** *p* < 0.001, ** *p* < 0.01, * *p* < 0.05.)

The result of the KEGG analysis revealed that HIF-1α/ Bcl-2/adenovirus E1B 19-kDa-interacting protein (BNIP3) signaling pathway, one of the autophagy pathways, was closely related to the role of HSYA in regulating autophagy. By analyzing the results of the WB assay, we observed that HSYA activated the expression of HIF-1α and BNIP3 after OGD/R, accompanied by an increase in LC3 and Beclin-1 expression and a decrease in P62 expression, indicating that HSYA might regulate the autophagy-related HIF-1α/BNIP3 pathway. To further validate this hypothesis, YC-1, an inhibitor of HIF1A, was added in the following experiment. It was found that with the inhibition of HIF1A, the activation of autophagy induced with HSYA was diminished, as shown by the decrease in LC3 and Beclin-1 expression and the increase in P62 expression (Figure 7a). In addition, the TUNEL assay showed that the number of apoptotic cells was significantly increased in SH-SY5Y cells silenced with HIF1A (Figure 7b), indicating that apoptosis is exacerbated by the inhibition of autophagy in SH-SY5Y cells in the acute phase of OGD/R. Taken together, we finally confirmed that HSYA exerts neuroprotective effects by regulating autophagy, which should be mediated through the HIF-1α/BNIP3 signaling pathway.

## 4. Discussion

Neuronal damage caused by IS is the leading cause of physical disability and even death in stroke patients. Its effective prevention and treatment remain a challenge, and therefore detailed studies have focused on this complex disease [16,17]. In this study, we screened and identified 61 relevant target genes by analyzing microarray samples from MCAO rats and finally obtained 10 IS-DEGs as core genes. GO and KEGG analyses revealed that these genes are mainly enriched in immune response, inflammatory response, apoptosis, the HIF-1 signaling pathways, and the PI3K-Akt signaling pathway. HIF1A and CASP3 pathways, as the stroke-related essential genes, are involved in autophagy and apoptosis after cerebral ischemic injury [18,19]. In addition, based on the results of network pharmacology combined with the related literature [20], we found that HSYA intervention induced HIF1A expression and reduced CASP3 expression in OGD/R-injured SH-SY5Y cells. This may be due to the activation of the transcription factor HIF-1α and its related signaling pathways by HSYA, which exerts a neuroprotective effect and thus attenuates neuronal apoptosis in ischemic–hypoxic injury [21].

Autophagy, one of the hot spots in biomedical research, is a lysosome-mediated degradation process of cellular components, which can be induced by nutrient depletion or I/R damage [22]. When the number of damaged organelles increases, external pathogens invade, or proteins accumulate abnormally, cellular contents are wrapped in vesicle membranes to form autophagosomes, which are further bound to lysosomes to form autolysosomes [23]. Nowadays, it is controversial whether autophagy induced by cerebral I/R has neuroprotective effects or induces injury [24]. Some studies have shown that autophagy is detrimental during neuronal ischemic–hypoxic injury [25,26], while others have shown that the activation of autophagy under ischemic conditions not only provides energy by degrading proteins but also protects cells from death [27,28]. Taken together, there are three factors which determine the autophagic function, namely, the activation level, the induction time, and whether autophagy flux is impaired. A previous study showed that the effects of autophagy are different at different stages of the disease, which was confirmed by autophagy antagonist 3-MA administered at different periods. Autophagy was activated within 24 h after cerebral I/R, and neuronal apoptosis was attenuated. However, when 3-MA was given 48–72 h after cerebral I/R, autophagy was inhibited, and cells were instead protected from death [29], indicating that autophagy is overactivated, resulting in a shift from protective to destructive effects as the duration of cerebral I/R extends. The present study focused on the time window of 12–24 h of OGD/R and showed that the activation of autophagy was adequate and timely and that within 24 h it exhibited a protective effect. In addition, whether autophagy flux is impaired can determine the function of autophagy [30], since in the present study autophagy flux was not disrupted and was upregulated. Based on these results, our study suggests that autophagy has a protective effect, which may be related to early activation of autophagy and unimpaired autophagic flux. 

Moreover, autophagy and apoptosis have been proved to be interrelated. There is strong evidence that autophagy attenuates the apoptotic cascade response in IS [31,32], and if autophagy is inhibited, further apoptosis may be occurring [33,34]. Based on the results of network pharmacology, the action of HSYA on CASP3 may play an essential role in treating IS. As an activated form of CASP3, Cleaved-Caspase-3 is a recognized marker of apoptosis [35]. Here, we hypothesized that the potential mechanism of HSYA anti-apoptosis might be due to the activation of autophagy. The changes in apoptosis induced by the administration of HSYA or autophagy inhibitors were observed in the present study. We confirmed that SH-SY5Y cells had increased apoptosis after OGD/R, and their autophagy levels were not enough to mitigate apoptosis. In contrast, HSYA further increased autophagy after OGD/R, accompanied by a decrease in apoptosis. However, the anti-apoptotic effect of HSYA was reversed after the treatment of the autophagy inhibitor 3-MA. Based on these results, we suggest that the induction of autophagy is involved in the protective mechanism of HSYA against neuronal OGD/R injury. HSYA acts as an anti-apoptotic factor in our in vitro model.

Interestingly, Zhang, Y. et al. [36] found that HSYA could inhibit autophagy through the HIF-1α/BNIP3/Notch-1 signaling pathway, and thus exert a cerebral protective effect, by establishing a middle cerebral artery occlusion–reperfusion (MCAO/R) rat model and treating it with HSYA for 3 days. This is inconsistent with our results and may be related to the herbal monomer’s multi-target and multi-pathway overall coordination. Therefore, we suggest that HSYA plays a protective role by activating autophagy in the acute phase of cerebral ischemia and, similarly, by inhibiting autophagy in the recovery phase, providing a new idea for our subsequent study. Our research aimed to understand the potential mechanism of HSYA in the treatment of ischemic stroke. The neuronal single-cell OGD/R model is helpful to control variables and reduce the interference of other factors except neurons. However, stroke is a complex pathophysiological process, and neuronal protection is affected by the microenvironment. Therefore, we plan to develop an HSYA treatment in vivo model with cerebral ischemia, providing sufficient experimental evidence for better application in clinical practice.

## 5. Conclusions

In summary, HSYA intervention further promoted the expression of HIF1A and inhibited the level of CASP3, accompanied by an increase in autophagy and a decrease in apoptosis in SH-SY5Y cells with OGD/R. The inhibition of HIF1A diminished the activation of autophagy induced with HSYA, while the inhibition of autophagy increased cell apoptosis and blocked the neuroprotective effect of HSYA, suggesting that the neuroprotective effect of HSYA should be mediated by activating the HIF1A/BNIP3 signaling pathway to induce autophagy.

## Data Availability

The datasets presented in this study can be found in online repositories. The names of the repositories and the accession numbers can be found in the GEO database with the following Accession Number: GSE100235.

## Supplementary Materials

The following supporting information can be downloaded at: https://www.mdpi.com/article/10.3390/cells11233726/s1, Figure S1: SH-SY5Y cells were treated with 6 h of OGD followed by 12 h of oxidation under HSYA intervention, Figure S2: The autophagic activation after HSYA intervention was accompanied by the survival of SH-SY5Y cells.
