# Supplementary figures and images for: Hydroxysafflor Yellow A Exerts Neuroprotective Effects via HIF-1α/BNIP3 Pathway to Activate Neuronal Autophagy after OGD/R
Source: Cells. 2022 Nov 22;11(23):3726. doi: 10.3390/cells11233726 (PMC9736542; doi:10.3390/cells11233726)

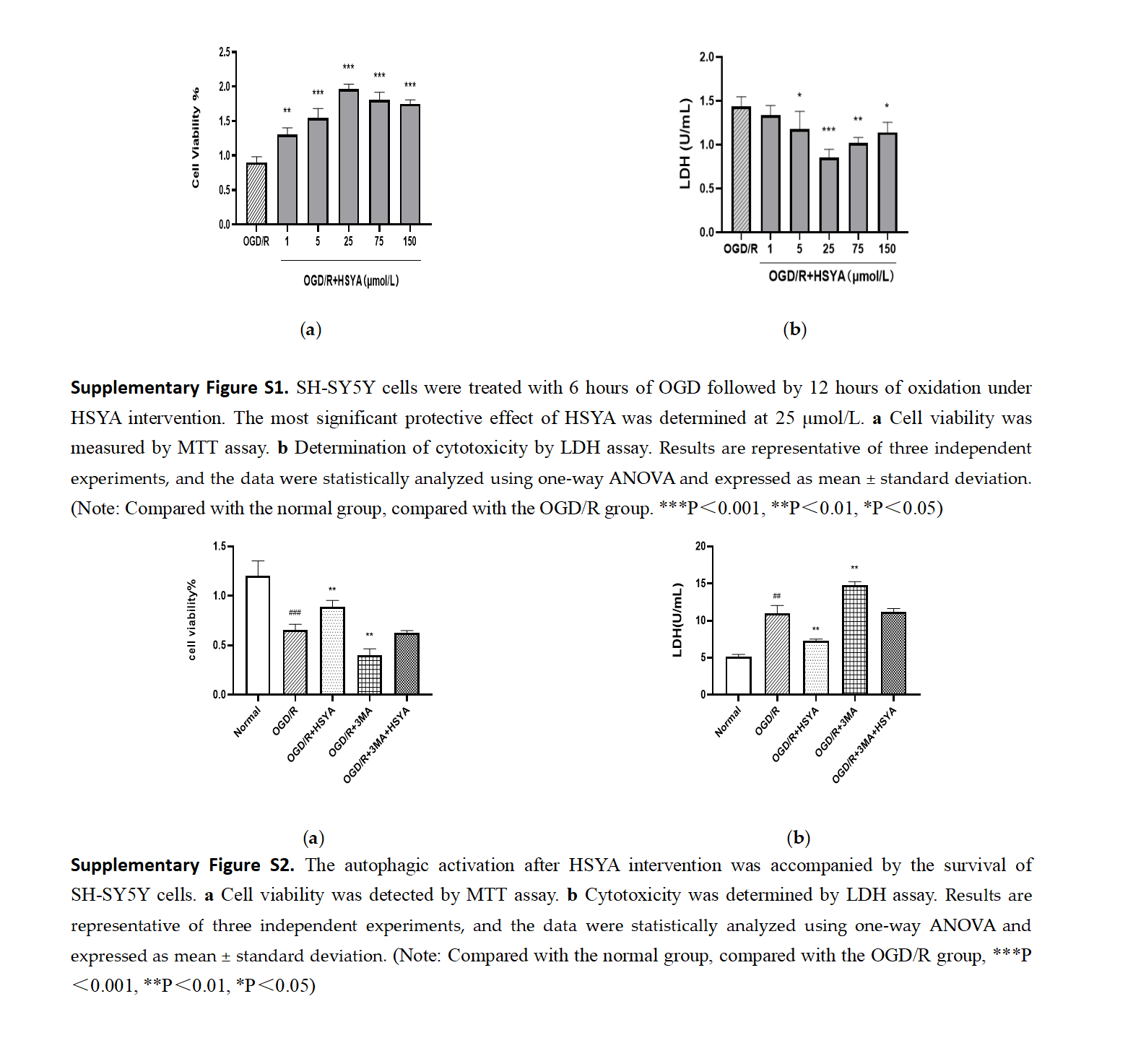

Supplement: Supplementary file 1 [file cells-11-03726-s001.zip › cells-1993764-supplementary/cells-1993764-supplementary.tif]
